# Role of cytokines in the interplay between cancer cells and stroma-associated monocytes

**DOI:** 10.1186/1471-2164-15-S2-P16

**Published:** 2014-04-02

**Authors:** Taoufik Nedjadi, Eithne Costello

**Affiliations:** 1 King Fahd Medical Centre, 21589 Jeddah, Kingdom of Saudi Arabia; 2 Department of Molecular and Clinical Cancer Medicine, The University of Liverpool, Liverpool L69 3GA, UK

## Background

Pancreatic cancer is characterized by the presence of a highly reactive stroma [1]. The latter harbors a variety of cellular compartments including stellate cells, fibroblasts, endothelial cells and a variety of inflammatory cells such as macrophages and monocytes. Interaction between tumour cells and surrounding stromal cells (tumour micro- environment) plays an important role in pancreatic cancer progression [2]. We have previously shown that stroma-associated monocytes express low molecular weight proteins: S100A8 and S100A9 [3]. The aim of this study is to investigate the involvement of S100A8 and S100A9 proteins in the tumour-stroma crosstalk and to decipher the potential signaling mechanism.

## Materials and methods

Cell culture of pancreatic cancer cells and monocytic cells, HL-60 and primary isolated human monocytes. Isolation of Conditioned medias from pancreatic cancer cells. Western blotting analysis. Cytokines multiplexing assay (27-plex, BioRad). Cell signaling assay. Luciferase assay.

## Results

1- The expression of S100A8 and S100A9 in monocytes is increased in response to soluble factors in pancreatic cancer cells conditioned media.

2- Cytokine profiling of cell supernatants, using Luminex assay, showed that PCC secrete a number of cytokines and growth factors including IL-8, FGF and TNF-α.

3- S100A8 and S100A9 increased phosphorylation of MAPK, erk1/2 and p38 and activated NF-kB signaling pathways in a RAGE dependent manner.

## Conclusions

S100A8 and S100A9 promote specific cytokine secretion from pancreatic cancer cells. Interestingly, a number of these cytokines, in turn, induce the secretion of S100A8 and S100A9 from monocytic cells, creating a paracrine loop. These events may create a favourable environment for tumour development and metastases.
